# Antifungal, antibacterial, and anti-inflammatory activity of glycerol dithionomonolaurate, an analog of glycerol monolaurate

**DOI:** 10.1128/msphere.00318-25

**Published:** 2025-10-02

**Authors:** Patrick M. Schlievert, Paul E. Brennan, Robert E. Klem, Dayton T. Reardan

**Affiliations:** 1 Niche Biopharmaceuticals, Shorewood, Minnesota, USA; 2 Nuffield Department of Medicine, Centre for Medicines Discovery, University of Oxford 105596, Oxford, United Kingdom; University of Nebraska Medical Center College of Medicine, Omaha, Nebraska, USA

**Keywords:** *Candida auris*, gram-positive bacteria, gram-negative bacteria, antimicrobial agent, anti-inflammatory, *Candida albicans*

## Abstract

Glycerol dithionomonolaurate (NB2) is an analog of the natural antimicrobial compound glycerol monolaurate (GML) with enhanced antimicrobial activity. The advantage of NB2 over GML is the presence of a dithionate to greatly reduce microbial cleavage through glycerol ester hydrolases, such as *Staphylococcus aureus* lipase. NB2 exhibited antimicrobial activity against *Candida* fungal species, including *Candida auris*, with comparable minimum bactericidal concentration (MBC) and minimum inhibitory concentration (MIC) of 50–100 µg/mL compared to 250–500 µg/mL for GML. NB2 exhibited a broad range of antibacterial activity including both gram-positive and gram-negative organisms, while sparing normal microbiome lactobacilli. When 10^8^ *C. auris* and 10^9^ *S. aureus* were incubated on Todd Hewitt agar plates in the presence of two times the MBC for NB2, no resistant colonies grew, likely the result of the large number of antimicrobial targets inhibited. Like GML, NB2 inhibited exotoxin production at non-antibacterial concentrations. NB2 exhibited anti-staphylococcal activity when tested topically on a dermatitis skin model on rabbits. NB2 inhibited the production of chemokines (interleukin-8 and macrophage inflammatory protein-3α) by human vaginal epithelial cells, suggesting anti-inflammatory activity. Unlike clear GML solutions, NB2 was pale yellow. The absorption spectrum of NB2 was not sufficient to raise Food and Drug Administration concerns over phototoxicity. Collectively, the data suggest that NB2 could become a useful topical agent for the prevention of both fungal and bacterial infections as a dual-active anti-infective, killing microbes and reducing harmful inflammation, such as seen in atopic dermatitis.

**IMPORTANCE:**

Fungi and many bacteria commonly develop resistance to antimicrobial agents or have inherent resistance. Many microbes initiate infections through the skin and mucous membranes, in part by producing toxins and causing harmful inflammation. We describe a novel topical antimicrobial agent, glycerol dithionomonolaurate (NB2), effective against *Candida* and a wide range of gram-positive and gram-negative bacteria; the compound did not kill normal microbiome lactobacilli. NB2 is likely to have many microbial targets for killing, suggesting resistance to the molecule may not develop. At sub-antibacterial concentrations, glycerol dithionomonolaurate inhibited exotoxin production by *Staphylococcus aureus*. The molecule was not inactivated by staphylococcal lipase. Glycerol dithionomonolaurate effectively treated *S. aureus* dermatitis in a rabbit skin model and reduced chemokine production by human epithelial cells. Glycerol dithionomonolaurate may prove useful to treat many types of skin and mucous membrane infections by both antimicrobial and anti-inflammatory activities, such as in atopic dermatitis.

## INTRODUCTION

Glycerol dithionomonolaurate (NB2) is a pale-yellow analog of glycerol monolaureate (GML, monolaurin). GML is broadly antimicrobial, killing gram-positive bacteria, gram-negative bacteria with a non-*Enterobacteriaceae* lipopolysaccharide or lipo-oligosaccharide, mycobacteria, mycoplasma, and fungi including *Candida* species, dermatophytes, and saprophytic fungi (1, 2). GML is also effective in preventing enveloped viral infections of cells (3), and in a non-aqueous gel is effective in preventing simian immunodeficiency virus (SIV) infection of rhesus macaques (4, 5). In a small pilot study, GML non-aqueous gel was effective in reducing vaginal *Gardnerella vaginalis* as a marker for bacterial vaginosis and in reducing *Candida* (2). In a phase 2 clinical trial, GML non-aqueous gel reduced *Candida* vaginally, increased vaginal lactobacilli, but did not reduce *G. vaginalis* (6). The reason for the increased vaginal lactobacilli after GML non-aqueous gel treatment is that many lactobacilli have an immunity gene against GML, present in strains because many lactobacilli produce a quorum-sensing growth stimulant analog of GML, called reutericyclin (7). *In vitro* studies confirm that GML does not kill lactobacilli (8).

Human safety is paramount in new drug development, and chronic safety studies, as performed for 6 months with twice daily (3 months) and then once daily (3 months) administration (1 mL each time) vaginally to rhesus macaques, showed that 50,000 µg/mL of GML in a non-aqueous gel (variant of K-Y Warming Gel) was safe (8). Additionally, a safety study performed in humans with 3 months of once-daily administration of 5 mL of the same gel indicated that the GML non-aqueous gel was safe. There were no serious adverse events noted in the pilot vaginal study in women to treat bacterial vaginosis and *Candida* infection, but there were minor adverse events noted in the phase 2 clinical trial (e.g., some women reported a stinging sensation vaginally upon initial application) (6). Finally, human nasal administration of GML non-aqueous gel indicated no adverse events in a 2-day application period, but with a significant reduction of *Staphylococcus aureus*, which lasted as long as 3 days (9). Thus, it was concluded that the 50,000 µg/mL GML non-aqueous gel was safe for use, increased mucosal normal flora lactobacilli, and killed pathogens (*Candida* and *S. aureus*).

The major drawback in the use of antimicrobials is the development of resistance in the microbes. We attempted to develop resistance to GML alone, and after a year of unsuccessful attempts, we concluded that there were too many GML targets in microbes for resistance to develop (1).

Another drawback we observed for the use of GML is that some microbes produce lipases (glycerol ester hydrolases) (10). Thus, if enough bacteria were present in an infection site (or test tube), additional GML could be needed to kill the pathogens due to GML cleavage and partial inactivation. GML is cleaved by glycerol esterases into non-antimicrobial glycerol and partially antimicrobial lauric acid (400 times less active than GML) (1). With this in mind, we made an analog of GML, glycerol dithionomonolaurate, which we named NB2. NB2 is the 2-sulfur dithionate analog of the oxy-ester containing GML. Our hypothesis was that the presence of the dithionate, due to the larger size of sulfur, may prevent inactivation by microbial lipases. This investigation evaluates NB2, compared to GML, for antimicrobial and anti-inflammatory activity both *in vitro* and *in vivo* in a rabbit infection model.

## RESULTS

### Properties of NB2 compared to GML

**Fig 1:**
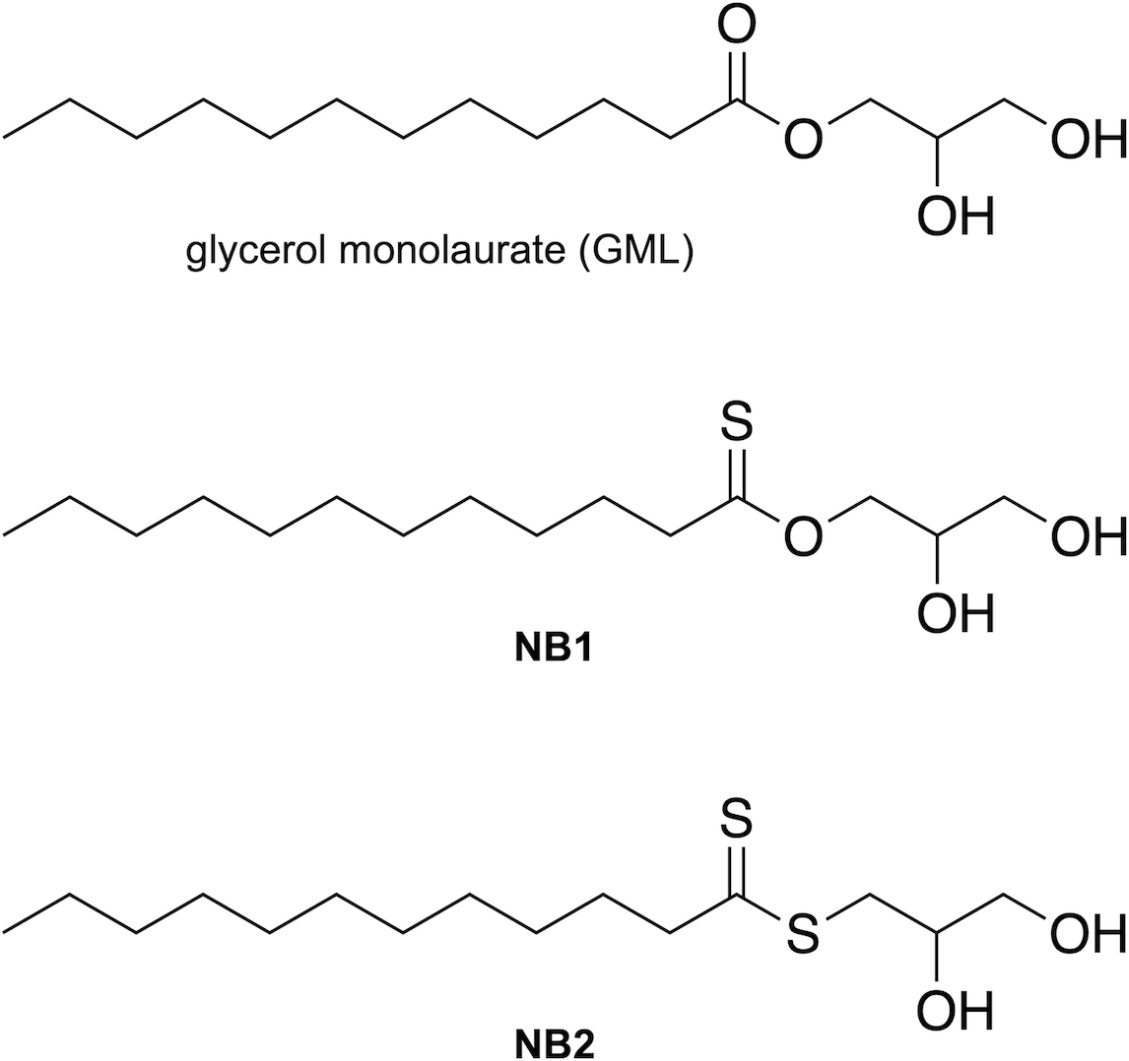
Chemical structures of GML, NB1 (thioate), and NB2 (dithioate). All compounds are racemic mixtures.

The structures of a control molecule for our studies, *O*-glyceryl dodecanethioate (NB1), glycerol dithionomonolaurate (NB2), and analog glycerol monolaurate (GML) are shown in Fig. 1. NB2 gives a pale-yellow solution compared to the colorless NB1 and GML when the compounds are added to absolute ethanol at 100,000 µg/mL. None of the three compounds is highly soluble in aqueous solutions (maximum 100 µg/mL at 37°C), whereas all three are easily soluble in ethanol.

**Fig 2:**
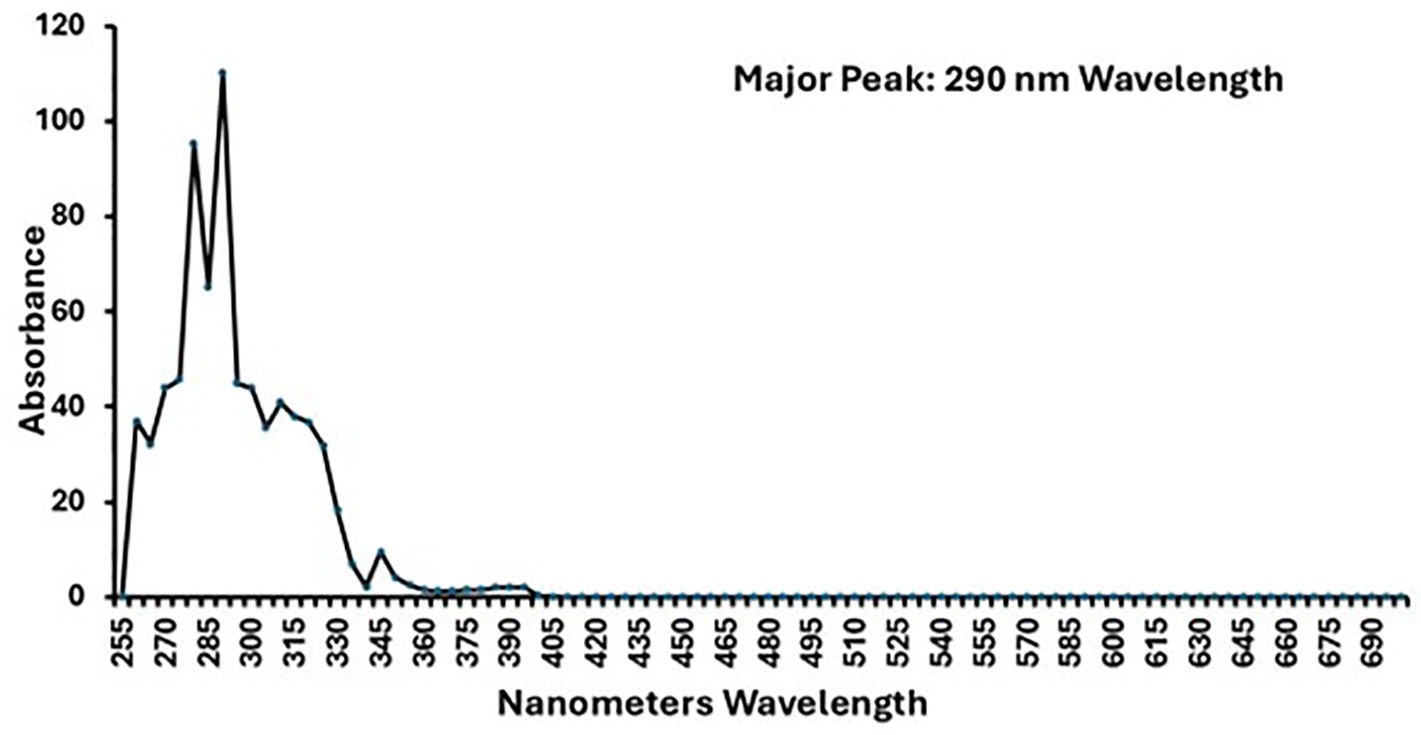
Absorbance spectrum of NB2 versus nanometers wavelength. NB2 was dissolved at 100,000 µg/mL in absolute ethanol. The compound in ethanol has a pale-yellow color.

We determined the absorbance spectrum of NB2 in absolute ethanol. NB2 had absorbance spectrum peaks at 280 and 290 nm wavelength (Fig. 2). The molar extinction coefficient of NB2 at the 290 peak was determined to be 340.9 L/mol/cm. This value is below the threshold set by the Food and Drug Administration for concerns of phototoxicity (1,000 L/mol/cm). Both NB2 and GML are soluble in ethanol and Vaseline at concentrations of 50,000 µg/mL as most often used in the present studies. In all experiments, NB2 maintained its pale yellow color, whereas NB1 and GML were clear. NB1, NB2, and GML were stored lyophilized as solids. NB2 was a yellow powder that showed no evidence of degradation (acquisition of a sulfur smell) even after nearly a year of storage. NB1 was a white powder with no smell, and GML was waxy white pellets with no smell.

### Synthesis of *O*-glyceryl dodecanethioate (NB1) and glycerol dithionomonolaurate (NB2)

**Fig 3:**
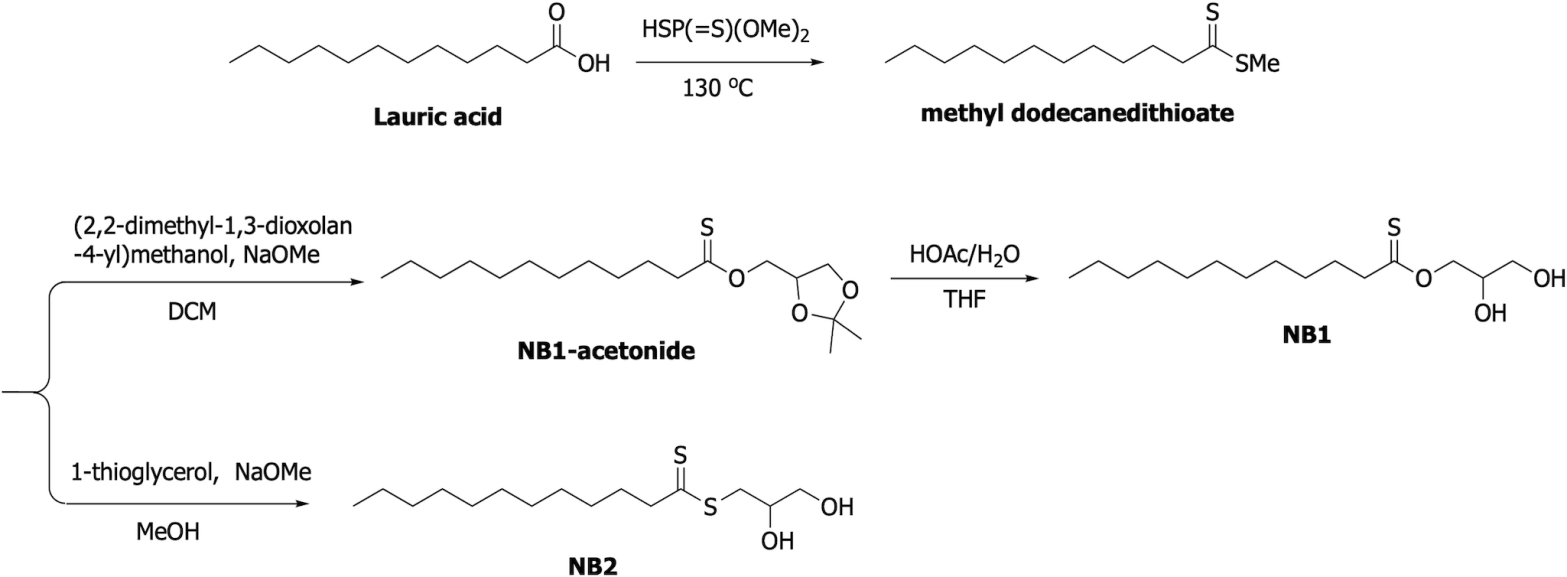
Summary of synthesis of NB1 and NB2 from lauric acid.

The overall synthesis of NB1 and NB2 is summarized in Fig. 3. Specific synthesis of both compounds is given below.

#### Methyl dodecanedithioate

Lauric acid (1.00 g, 5 mmol) was added to *O*,*O*-dimethyl *S*-hydrogen phosphorodithioate (5.0 mL) under N_2_. The reaction mixture was heated to 130°C for 1.5 h. It was cooled to room temperature. Diethyl ether (50 mL) was added to it, and it was washed with saturated NaHCO_3_ (25 mL ×3) and saturated sodium chloride (30 mL), then dried over Na_2_SO_4_. It was concentrated by evaporation under reduced pressure. The residue was purified by silica column chromatography (petroleum ether) to give methyl dodecanedithioate (0.57 g, 46%). ^1^H NMR (300 MHz, CDCl_3_) δ 3.04 (m, *J* = 7.5 Hz 2H), 2.62 (s, 3H), 1.85–1.78 (m, 2H), 1.37–1.26 (m, 16H), and 0.88 (t, *J* = 7.2 Hz, 3H).

#### NB1-acetonide

(2,2-Dimethyl-1,3-dioxolan-4-yl) methanol (0.45 mL, 3.60 mmol) was dissolved in dichloromethane (11 mL) containing sodium methoxide (0.19 g, 3.60 mmol). Methyl dodecanedithioate (0.59 g, 2.40 mmol) was added and the solution was stirred (under N_2_) at room temperature for 0.5 h. It was concentrated by evaporation under reduced pressure. Diethyl ether (60 mL) was added to it, and it was washed with water (15 mL) and saturated sodium chloride (20 mL ×2), then dried over Na_2_SO_4_. It was concentrated by evaporation under reduced pressure. The residue was purified by silica column chromatography (Petroleum ether:Ethyl acetate = 90:1–60:1) to give NB1-acetonide (115 mg, 14%). ^1^H NMR (300 MHz, CD_3_OD) δ 4.51–4.11 (m, 3H), 4.15–4.10 (m, 1H), 3.82–3.77 (m, 1H), 2.75 (t, *J* = 7.5 Hz, 2H), 1.80–1.70 (m, 2H), 1.41 (s, 3H), 1.35 (s, 3H), 1.30 (m, 16H), and 0.90 (t, *J* = 6.9 Hz, 3H).

#### NB1

To a solution of NB1-acetonide (0.382 g, 1.15 mmol) in tetrahydrofuran (6.5 mL) was added acetic acid aqueous solution (8.18 mL, 60%) under N_2_. The reaction mixture was heated to 120°C for 1.5 h. It was cooled to room temperature. Diethyl ether (100 mL) was added to it, and it was washed with water (30 mL ×2), saturated NaHCO_3_ (30 mL ×3), and saturated sodium chloride (20 mL ×2), then dried over MgSO_4_. It was concentrated by evaporation under reduced pressure. The residue was purified by silica column chromatography (Petroleum ether:Ethyl acetate = 3:2) to give NB1 (100 mg, 29.8%). ^1^H NMR (300 MHz, CD_3_OD) δ 3.83–3.79 (m, 1H), 3.57–3.50 (m, 3H), 3.26 (d, *J* = 7.5 Hz, 1H), 3.03 (t, *J* = 7.2 Hz, 2H), 1.88–1.78 (m, 2H), 1.39–1.29 (m, 16H), and 0.90 (t, *J* = 6.6 Hz, 3H).

#### NB2

1-Thioglycerol (0.26 mL, 2.95 mmol; freed of water by azeotropic distillation with toluene) was dissolved in absolute methanol (19.4 mL) containing a catalytic amount of sodium methoxide (16 mg, 0.30 mmol). Methyl dodecanedithioate (0.87 g, 3.54 mmol) was added and the solution was stirred at room temperature for 1.5 h. Diethyl ether (100 mL) was added to it, and it was washed with water (20 mL) and saturated sodium chloride (40 mL), then dried over MgSO_4_. It was concentrated by evaporation under reduced pressure. The residue was purified by silica column chromatography (Petroleum ether:Ethyl acetate = 3:2) to give NB2 (89 mg, 19%). ^1^H NMR (300 MHz, CD_3_OD) δ 3.83–3.79 (m, 1H), 3.57–3.50 (m, 3H), 3.26 (d, J = 7.5 Hz, 1H), 3.03 (t, J = 7.2 Hz, 2H), 1.88–1.78 (m, 2H), 1.39–1.29 (m, 16H), and 0.90 (t, J = 6.6 Hz, 3H).

NB2 was purified by high-pressure liquid chromatography (Waters Acquity UPLC HSS C18 SB 1.8 um, 2.1 × 50 mm column, Part NO. 186004118); 4.5 min; gradient 5–95% MeCN/H_2_O (0.1% formic acid), 0.5 mL/min. Purity was 99.9%.

### *In vitro* growth experiments

**Fig 4:**
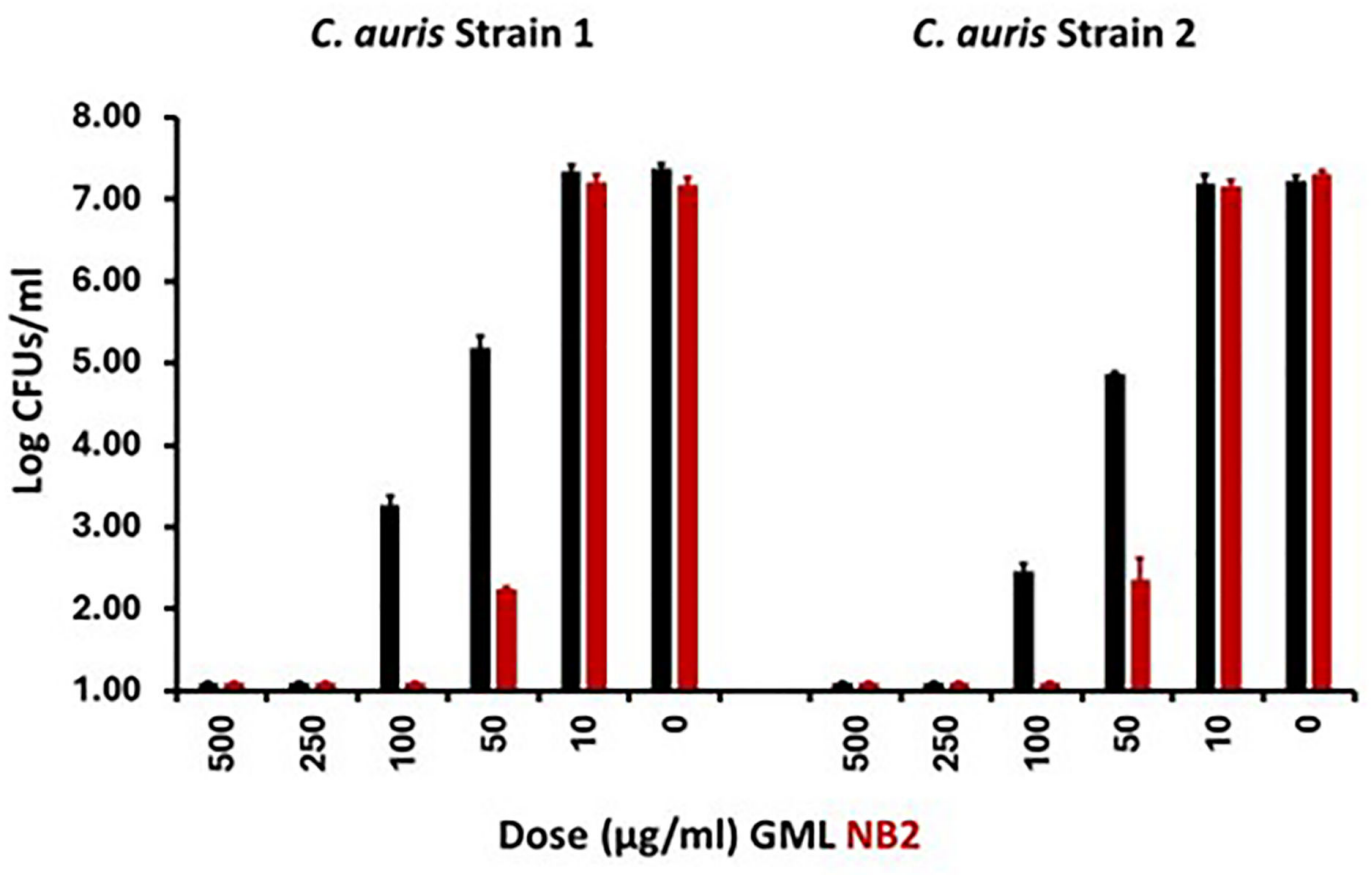
Effect of glycerol dithionomonolaurate (NB2) and glycerol monolaurate (GML) on 24 h growth of two recent clinical isolates of *Candida auris*. Inoculum size in Todd Hewitt broth was 10^5^/mL. Cultures were incubated at 37°C with 200 revolutions/min shaking. Plate counts were used to assess CFUs/mL. CFUs were log_10_ transformed prior to graphing. A greater than 3-log_10_ drop in CFUs/mL from the inoculum was considered bactericidal. CFUs/mL with <1 log_10_ increase but <3 log_10_ drop in CFUs/mL, compared to the inoculum, was considered bacteriostatic.

All *in vitro* and *in vivo* experiments have been performed a minimum of two times. Initial *in vitro* experiments were performed with *Candida auris* (Fig. 4). We used inoculum doses of both *C. auris* strains consistent with the *C. albicans* numbers that may be present vaginally during menstruation (up to 10^5^ CFUs/mL in vaginal secretions) (2, 6). We evaluated the killing of two *C. auris* clinical isolates. The minimum bactericidal concentrations (MBCs) of NB2 and GML were considered a ≥3 log reduction in CFUs/mL compared to the no antimicrobial agent control. The MBC of NB2 for both *C. auris* strains was 50 µg/mL, compared to 100 µg/mL for GML.

**Fig 5:**
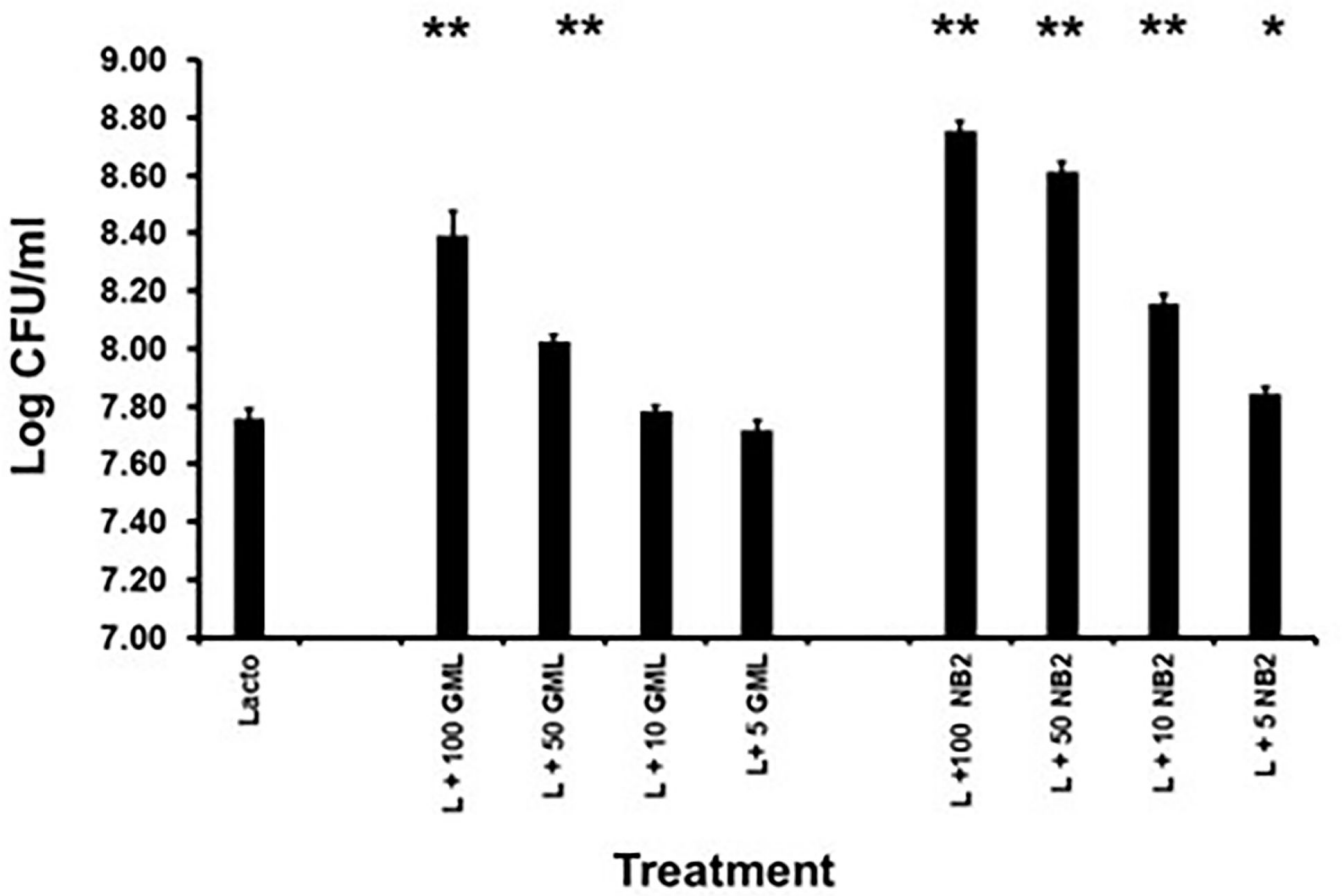
Effect of glycerol dithionomonolaurate (NB2) glycerol monolaurate (GML) on the 48 h growth of *Lactobacillus crispatus* (Lacto, L). Amounts of NB2 and GML are in µg/mL. Starting inoculum was approximately 10^6^/mL. *P < 0.0.05 and **P < 0.001 compared to lactobacillus alone.

The antimicrobial spectrum of NB2 was also compared to GML for many of the same microbes studied previously for GML (1). NB2 killed both gram-positive bacteria and gram-negative bacteria including gram-negative bacteria that were not killed by GML (Table 1). Gram-positive cocci were killed at approximately four times lower NB2 concentration than needed for GML. The one exception is that neither NB2 nor GML killed *Lactobacillus crispatus*, a member of the normal microbiome (Fig. 5). This was considered a favorable outcome, as maintaining healthy dermal flora is desirable.

**TABLE 1:** Minimum bactericidal concentration of glycerol dithionomonolaurate (NB2), compared to glycerol monolaurate (GML) for *Candida species* and gram-positive and gram-negative bacteria

Microbe	Isolate number	Minimum bactericidal concentration for:	
		NB2 (µg/mL)	GML (µg/mL)
Fungi			
*Candida auris*	2	50	100
*Candida albicans*	10	50	100
*Candida glabrata*	1	50	100
*Candida krusei*	1	50	100
Gram-positive bacteria			
*Staphylococcus aureus* (MRSA)	5	60	500
*Staphylococcus aureus* (MSSA)	3	60	500
*Streptococcus pyogenes* (Group A)	4	5	30
*Streptococcus agalactiae*	4	5	30
*Clostridioides difficile*	2	5	25
Gram-negative bacteria			
*Haemophilus influenzae* (nontypable)	1	30	250
*Gardnerella vaginalis*	1	3	15
*Escherichia coli*	2	500	>5,000
*Salmonella* Minnesota (wild type)	1	250	2,000
*Salmonella* Minnesota (Re mutant)	1	5	25
*Klebsiella pneumoniae*	1	500	>5,000

NB2 killed all gram-negative bacteria tested more effectively than GML (Table 1). GML alone does not kill *Enterobacteriaceae*, unless the intact lipopolysaccharide is removed (10). Thus, in that prior study, GML effectively killed *Salmonella* Minnesota Re mutant (lacking the O-specific side chain and common core), but GML alone killed wild-type *Salmonella* Minnesota at 2,000 µg/mL or greater. In a prior study, GML alone was effective in killing gram-negative bacteria with a lipo-oligosaccharide, such as *G. vaginalis* and *Haemophilus influenzae* (1, 2).

NB2 was also effective in killing other species of *Candida,* including *Candida albicans*, *Candida glabrata,* and *Candida krusei*. For all microbes with susceptibility to NB2, the agent was two times as effective at killing microbes as GML. These amounts of GML to kill *Candida species* are generally in agreement with our prior findings (2).

In one additional experiment for antimicrobial activity, we incubated in triplicate on TH agar plates 10^8^ *C. auris* and 10^9^ *S. aureus*, incorporating two times the MBC of NB2 or GML in the plates for 24 and 48 h. No resistant colonies of either microbes grew in the presence of either agent. We had previously shown that even incubation for 1 year with weekly passages on reduced GML amounts, no increase in MBC was observed for *S. aureus* when plated onto the MBC (1). This was thought to occur because of the large number of membrane targets of GML, contributing to antimicrobial activity. The same was hypothesized for NB2 since it is an analog of GML. NB2 and GML were 48 h quorum growth stimulants for *L. crispatus* (Fig. 5), like our *in vivo* (humans) observation in a phase 2 clinical trial with GML (6). NB2 was more potent than GML in this activity.

We tested NB2 for ability to inhibit TSST-1 production by *S. aureus* MN8, similar to what we observed for GML (10). NB2 was 50-fold more active at inhibition of TSST-1 production than it was at killing *S. aureus* (Table 2). *S. aureus* strains produce a potent glycerol ester hydrolase (lipase) which is able to hydrolyze and inactivate GML.

**TABLE 2:** Effect of glycerol monolaurate (GML) and glycerol dithionomonolaurate (NB2) on production of toxic shock syndrome toxin-1 (TSST-1) by *S. aureus^a^*

Agent	Dose	Log ± SD (CFUs/mL)	TSST-1 ± SD (µg/mL)
GML	125	**10.09 ± 0.07**	**<0.001**
	64	**10.21 ± 0.06**	**<0.001**
	32	**10.14 ± 0.06**	**5.0 ± 1.0**
	16	10.16 ± 0.01	23 ± 2.2
	8	10.17 ± 0.05	24 ± 3.4
	4	10.15 ± 0.03	20 ± 5.0
	0	10.15 ± 0.03	21 ± 2.2
NB2	125	1.0	<0.001
	64	3.51 ± 0.06	<0.001
	32	**10.10 ± 0.05**	**<0.001**
	16	**10.18 ± 0.02**	**<0.001**
	8	**10.12 ± 0.04**	**<0.001**
	4	**10.15 ± 0.05**	**14 ± 1.4**

^*a*^ Bolded numbers indicate inhibition of TSST-1 while not inhibiting *Staphylococcus aureus* growth.

### Lipase studies

**Fig 6:**
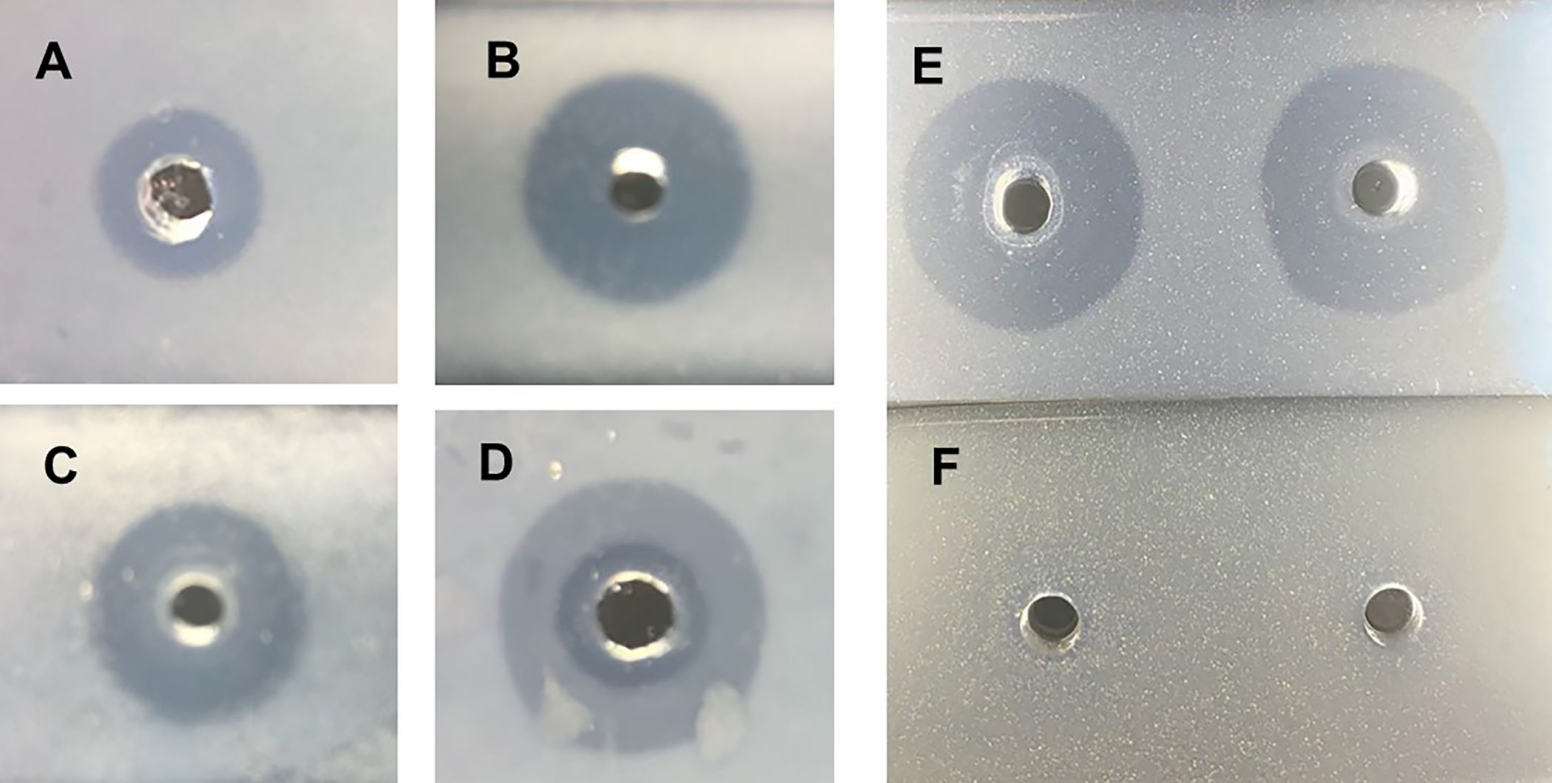
Hydrolysis of tributyrin, GML, NB1, and NB2 by *S. aureus* glycerol ester hydrolases (lipases). (**A**) Effect of purified *S. aureus* lipase, 0.1 µg/20 µL, on tributyrin (control); (**B**) effect of *Staphylococcus aureus* MN8 culture fluid lipase on tributyrin (control); (**C**) effect of purified lipase (0.1 µg/20 µL) on GML (control); (**D**) effect of *S. aureus* supernatant on GML; (**E**) effect of two different *S. aureus* (MN8 and MNPE) supernates on NB1 (control); and (**F**) effect of two different *S. aureus* (MN8 and MNPE) supernates on NB2. All compounds were suspended as insoluble mixtures in PBS-0.85% agarose. Clearing of the agarose with emulsified compounds after 24 h incubation at 37°C was considered evidence of staphylococcal lipase activity.

We tested NB2 and NB1, GML, and tributyrin (the latter three positive controls) for hydrolysis by supernatant fluids from *S. aureus* strains (Fig. 6). Tributyrin is the substrate we have used in prior studies of hydrolysis by staphylococcal lipase (11–13). Tributyrin was cleaved by both purified staphylococcal glycerol ester hydrolase (lipase) and by sterile supernatant fluid from growth of *S. aureus* MN8 in TH broth to stationary phase. GML is also susceptible to hydrolysis by staphylococcal lipase and was cleaved by MN8 culture fluid (Fig. 6). NB1 is a convenient control since it has one thiol, and it has an oxy-ester. NB1 was lysed by two supernatant fluids (*S. aureus* MN8 and MNPE), as evidenced by clearing of a microscope slide with embedded NB1 (Fig. 6). However, at the same concentration of NB2 as GML and NB1, NB2 was not hydrolyzed by the two supernatant fluids. It is unclear why Fig. 6D has two apparent zones of *S. aureus* MN8 lipase clearing of GML. It is possible that *S. aureus* MN8 contains another molecule responsible for the clearing.

### Human vaginal epithelial cell (HVEC) studies

**Fig 7:**
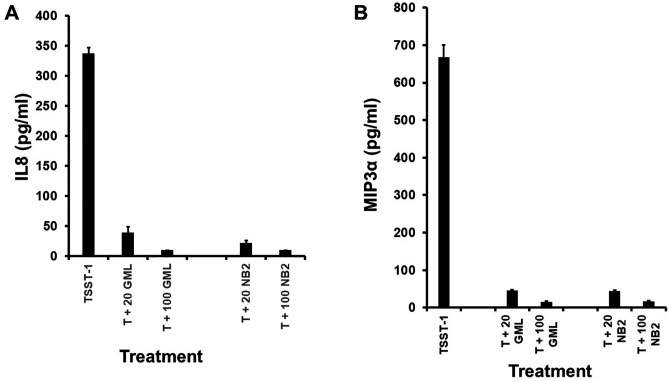
Effect of glycerol monolaurate (GML) and glycerol dithionomonolaurate (NB2) on production of (**A**) IL8 and (**B**) MIP3α by isolated human vaginal epithelial cells stimulated 6 h with 100 µg/well toxic shock syndrome toxin-1 (TSST-1, T). Cells were incubated in pyrogen-free keratinocyte serum-free medium at 37°C, 5% CO_2_. Doses of GML and NB2 were 20 and 100 µg/mL (20 and 100). Data represent means ± standard deviations. Means of cells exposed to lipopolysaccharide-free, highly purified TSST-1 versus cells exposed to TSST-1 plus 20 or 100 µg GML or NB2 were different with *P* < 0.001.

GML is not toxic to rhesus macaques or women when 50,000 µg/mL administered vaginally (1 mL to rhesus macaques or 5 mL to women) in a non-aqueous gel (4–6, 8), even over a 6-month time period, and as done with a safety study in rhesus macaques (8). NB2 and GML were not toxic *in vitro* to HVECs up to 100 µg/mL (>95% viable by Trypan blue dye exclusion after 6 h; Table 3). These data show that NB2 was equally non-toxic to HVECs as GML. At concentrations of NB2 and GML of 20 µL of 20 and 100 µg/mL, production of the chemokines IL8 and MIP3α, as induced in HVECs by TSST-1 (100 µg), was inhibited from being produced by HVECs, suggesting a promising effect on harmful pro-inflammation (Fig. 7). We have previously shown that pathogens, such as *S. aureus*, group A streptococci, and SIV, induce harmful inflammation initiated at mucosal and skin surfaces as a partial mechanism to contribute to disease causation (4, 5, 14, 15). We refer to this as outside-in signaling to cause infections (14). The limitation of these studies is that inhibition of inflammation was not tested *in vivo*.

**TABLE 3:** Trypan blue dye assessment of NB2 and GML toxicity to HVECs

Treatment	Dose (µg/mL)	Viability ± SD^*a*^ (%)
None		100 ± 0
NB2	20	100 ± 0
	100	97 ± 1.3
GML	20	100 ± 0
	100	96 ± 0.8

^*a*^ SD, standard deviation.

### Rabbit studies

**Fig 8:**
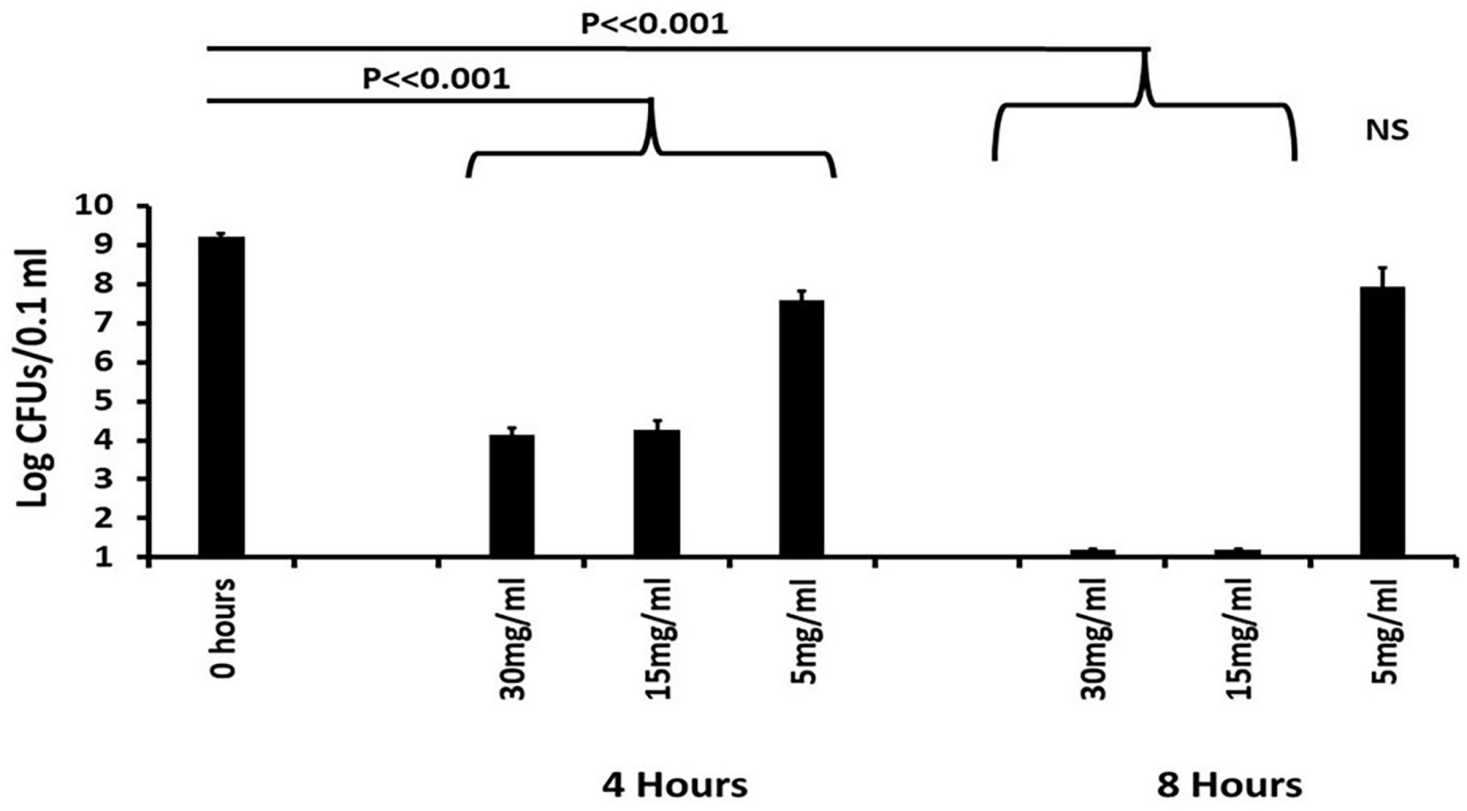
Effect of NB2 on *Staphylococcus aureus* MN8 in a rabbit dermatitis model. New Zealand white rabbits, male and female, had their flanks shaved. Then, 10^9^ *S. aureus* in pyrogen-free PBS (0.1 mL) was swabbed onto the flanks. Subsequently, the flanks were swabbed with 0.5 mL of NB2 in Vaseline. Swabs of the affected areas were taken at 4 and 8 h for determination of CFUs/0.1 mL. The CFUs were log-transformed for statistical analyses (Student’s *t*-test). Vaseline as a control had no effect on CFUs/0.1 mL.

Noting the difference between use of GML *in vivo* versus *in vitro* on HVECs, we tested the ability of NB2 to be non-toxic to New Zealand white rabbits, both sexes, and to inhibit the growth of 10^9^ dermal *S. aureus* MN8 (Fig. 8). We first painted the *S. aureus* on the shaved skin and then administered NB2 in Vaseline at NB2 concentrations of 5,000, 15,000, and 30,000 µg/mL. We used Vaseline only as the placebo control. Previously in similar experiments, we observed no toxicity of GML (16). Similar to that prior study, NB2 was not toxic to rabbits over the 8-h time point. The animals remained healthy in appearance, had no unusual swelling or reddening of the flank application sites, and appeared no different than Vaseline alone applied to the flanks. At both time periods (4 and 8 h), *S. aureus* was killed by NB2 at the two higher doses. The lower dose (5,000 µg/mL) reduced *S. aureus* numbers significantly at 4 h, but regrowth of the bacteria occurred by 8 h, so the effect was not significant. This experiment was repeated one additional time with similar results.

## DISCUSSION

This project was developed based on our many prior studies of glycerol monolaurate, and antimicrobial and anti-inflammatory agent with efficacy against bacteria, fungi, and enveloped viruses (1–5, 8, 10, 17). This novel compound, we designated NB2, is a thiol ester analog of GML. It was produced and tested because we hypothesized the new compound would not be degraded by esterases, which do have the ability to hydrolyze GML, for example, *S. aureus* lipase. Since GML and NB2 are designed for topical mucous membrane and skin use, where *S. aureus* and other pathogens commonly initiate diseases (4–6, 14, 18–20), we proposed NB2 may be more effective topically than GML.

NB2 has a mild yellow color and is odorless unless it degrades, whereupon it acquires a slight sulfur smell. The yellow color is insufficient to raise alarms about phototoxicity. NB2 has a similar broad spectrum of antimicrobial activity as GML, except NB2 also kills gram-negative wild-type *Enterobacteriaceae*, whereas GML does not kill that broad group. Usually, *Enterobacteriaceae* do not cause skin infections, but it is estimated that up to 4% of dermatitis is associated with this family of organisms. NB2 is also approximately four times more active against potential pathogens than GML. Neither NB2 nor GML kill lactobacilli, which are considered normal microbiome organisms on skin and mucous membranes. This positive attribute may depend on the presence of an immunity gene in certain lactobacilli as shown previously for GML and its analog reutericyclin (1, 7). There is no evidence to date of microbial resistance to GML and NB2, possibly because of the large number of presumed microbial membrane targets of the molecules. Both NB2 and GML inhibit exotoxin production at concentrations that do not kill the bacteria. Finally, both NB2 and GML are anti-inflammatory. Skin and mucous membrane pathogens initiate infections in part or in total through an outside-in signaling mechanism, whereby they cause harmful inflammation that opens the protective barrier (4, 14). Harmful inflammation occurs in part from IL8 and MIP3α stimulation of epithelial cells and keratinocytes (4, 5, 14, 15), the two chemokines we studied in this manuscript. Interleukin 8 (IL-8) attracts polymorphonuclear leukocytes (PMNs) to sites of pro-inflammation, and MIP3α attracts most other types of immune cells.

The most important attribute of antimicrobial agents should be their lack of significant toxicity at therapeutic doses. Extensive animal and human studies have shown GML is not toxic for use on mucosal and skin surfaces, recognizing that skin is more likely to be more impenetrable than mucous membranes (4–6, 8, 9, 16). Skin’s primary function is protection against external antigens as part of the innate immune system. Mucous membranes, in part, have a similar function, but they are not as strong a barrier since they have other secretory functions. Indeed, GML is generally recognized as safe as a food additive by the Food and Drug Administration. Because of those properties, we hypothesized NB2 would not be toxic to skin and mucous membranes. We thus began studies to assess NB2 toxicity and efficacy in a rabbit model of skin infection over an 8-h time period. NB2 was highly effective at 30,000 and 50,000 µg/mL at killing 10^9^ *S. aureus*. At the same time, the rabbits showed no sign of toxicity. For these studies, we used Vaseline as opposed to a K-Y Gel variant, since some women reported a vaginal stinging sensation upon application of GML in the variant K-Y Gel (6). Vaseline is a milder solubilizing agent.

It is important to consider that we used infection with 10^9^ *S. aureus* in the rabbit skin studies. Typically, persons with diabetes mellitus and atopic dermatitis are colonized and overtly infected with this organism (19, 20). However, the patients are usually colonized with up to 10^6^ *S. aureus* per the same body area as in our present study. Thus, patients are colonized with three logs less of the bacteria on the same body site. Our study in rabbits with a large dose of *S. aureus* was designed as a first step in *in vivo* experimentation, and we chose to use a large excess of bacteria.

Collectively, our studies show that NB2 may be a suitable topical antimicrobial agent, for example, in atopic dermatitis, with advantages over GML, including an enlarged spectrum of activity and more activity. Future studies will require additional preclinical testing, for example, absorption through skin. If successful, the downstream studies will include phase 1 and 2 human toxicity and efficacy studies.

## MATERIALS AND METHODS

### Bacteria and *Candida*

Bacterial and *Candida* strains are the same as previously published (1). Briefly, for *Candida* and *Staphylococcus,* we obtained two *C. auris* strains from the University of Iowa Hospitals and Clinics and multiple *C. albicans*, *C. glabrata*, and *C. krusei* strains from our −80°C laboratory stocks of human vaginal isolates of low passage. *S. aureus* strains included two of each clonal group of CC5 USA100 (IA116 and IA132), CC30 USA200 (MN8, MNPE), CC8 USA300 (LAC, LEVY), and CC1 USA400 (MNKN MNGN) strains which represented clinical isolates from patients with toxic shock syndrome (TSS) and/or sepsis (21). Strains IA116, IA132, LAC, MNKN, and MNGN were methicillin-resistant *S. aureus* (MRSA); the other strains were methicillin-sensitive *S. aureus* (MSSA). These organisms under low passage were stored in the laboratory at −80°C. All other microbes are as described previously (1).

### NB1, NB2, tributyrin, and GML assays for lipase degradation

NB1 and NB2 were synthesized as described in the Results section. We have extensively used tributyrin (glycerol tributyrate; ACROS Organics, Geel, Belgium) as a substrate for *S. aureus* glycerol ester hydrolase (lipase) activity (1, 11–13). Food grade GML was purchased from Colonial Chemical (South Pittsburg, TN, USA). Soluble stock solutions of NB1, NB2, and GML were prepared at 100,000 µg/mL in absolute ethanol. Tributyrin was used as purchased. Solutions of 0.85% agarose (Research Products International, Prospect, IL, USA) were prepared by heating in phosphate-buffered saline (PBS) (0.005 M NaPO_4_ and 0.15 M NaCl). As a positive lipase control, 10 mL of the soluble agarose solution was mixed with 0.1 mL of tributyrin. The solution was vortexed for 1 min to make a turbid suspension of the insoluble tributyrin in the agarose solution. Immediately, 4.5 mL of the suspension was added to standard microscope slides and allowed to cool. The slides were then stored at room temperature until used. For NB1, NB2, and GML, 0.1 mL of the solutions of 100,000 µg/mL, separately, were added to 10 mL of 0.85% melted agarose solution. The suspensions were vortexed for 1 min, and 4.5 mL was added to standard microscope slides. After solidifying, the slides were stored at room temperature until used.

At the time of the lipase assay, 4 mm wells were punched in the NB1, NB2, GML, and tributyrin agarose slides. To these wells, we added 0.1 µg/20 µL of purified staphylococcal lipase (prepared according to our standard toxin preparation [22] from *S. aureus* MN8) or 20 µL of sterile, stationary-phase supernatant fluids (centrifugation 10,000 × *g*, 10 min followed by filtration [0.2 µm pore size]) from 24 h growth of *S. aureus* MN8 or MNPE. The slides were incubated humidified for 24 h at 37°C in a 5% CO_2_ and then the slides were examined for clearing of turbidity. Clearing indicated esterase cleavage of NB1, NB2, GML, and tributyrin.

### *In vitro* microbial inhibition assays

Bacteria (except streptococci) were cultured at 37°C with shaking (200 revolutions per min) in Todd Hewitt broths (Difco Laboratories, Detroit, MI, USA). Group A streptococci (*Streptococcus pyogenes*), *H. influenzae, L. crispatus,* and *G. vaginalis* strains were cultured in Todd Hewitt broths stationary in the presence of 5% CO_2_. GML and NB2 (solubilized in absolute ethanol) were added to cultures with starting inoculums of 10^5^–10^7^/mL, depending on microbe. Cultures were incubated in triplicate for 24 h and then colony-forming units (CFUs/mL) were determined on sheep blood agar or chocolate agar plates. Two clinical isolates of *Clostridioides difficile* were cultured similarly with the same concentrations of GML and NB2, except the organisms were grown stationary in an anaerobic chamber for 48 h in pre-reduced TH broths. Plate counts were determined on chocolate agar plates.

In one set of studies, NB2 and GML, at two times the MBC, were incubated with 10^8^ or 10^9^ colony-forming units (CFUs) of *C. auris* or *S. aureus*, respectively, spread onto TH agar plates. After 24 and 48 h, the plates were examined for colonies resistant to NB2 or GML.

The ability of NB2 and GML to inhibit the production of the superantigen toxic shock syndrome toxin-1 (TSST-1), independent of effect on bacterial growth, was measured with use of NB2 and GML concentrations after 24 h growth of *S. aureus* strain MN8 (23), which did not inhibit growth. TSST-1 was quantified by a Western immunoblot procedure (24).

### *In vivo* antimicrobial activities

A total of 12 New Zealand white rabbits, both male and female (2–3 kg), were used in these studies. The studies were performed with ABSL-2 conditions under an approved protocol by the University of Iowa IACUC (Number 3072547-001). Rabbits were administered buprenorphine for pain management by a University of Iowa veterinary technician. Then, the animals had both flanks shaved. The animals subsequently received approximately 10^9^ CFUs of *S. aureus* strain MN8 in 0.1 mL of pyrogen-free PBS (0.005 M NaPO_4_, pH 7.2, 0.15 M NaCl) painted onto the exposed skin over a 4 cm × 4 cm area. Then, the same areas were painted one time with one of the following: NB2 in Vaseline or Vaseline alone. The animals were then returned to their cages and monitored for viable *S. aureus* at 4 and 8 h. For plate count determinations, the rabbits were swabbed diagonally across the infected area of exposed skin by rolling a PBS-wetted swab (holds 0.1 mL) one time. For the 4 h reading, the swab was rolled right to left, and for the 8-h reading, the swab was rolled left to right. At 8 h, the veterinary technician euthanized the animals. Plate counts were performed on sheep blood agar plates after making dilutions in Todd Hewitt broth followed by immediate plating.

### HVEC assays

The source and immortalization of HVECs has been extensively described by our laboratory (25, 26). HVECs were cultured in 96-well tissue culture plates in pyrogen-free keratinocyte serum-free medium (KSFM) until confluent at 37°C in the presence of 5% CO_2_. Subsequently, 100 µg of lipopolysaccharide-free, highly-purified superantigen TSST-1 (22) was added to wells in quadruplicate, except negative control wells. Then, various concentrations, in quadruplicate of NB2 or GML, were added to the wells that did or did not contain TSST-1. After 6-h incubation at 37°C in the presence of 5% CO_2_, IL-8 and macrophage inflammatory factor-3α (MIP3α) were measured in the supernatant fluids, as measures of effect on inflammation (Quantikine R&D Systems, Minneapolis, MN, USA). IL-8 is a chemokine that attracts PMNs to initiate inflammation at infection sites, and thus a measure of pro-inflammation. MIP3α attracts most other cells of the immune system to infection areas and thus also measures pro-inflammation. We did not assess the ability of NB2 and GML to inhibit lipopolysaccharide (LPS)-induced stimulation of HVECs since the HVECs lack responsiveness to LPS (25, 26).

### Statistics

Data in graphs were means ± standard deviations. Student’s *t*-test analysis was used to assess differences in means. All experiments were performed at least two times, including the studies in rabbits.
